# Towards Autonomous Agriculture: Automatic Ground Detection Using Trinocular Stereovision

**DOI:** 10.3390/s120912405

**Published:** 2012-09-12

**Authors:** Giulio Reina, Annalisa Milella

**Affiliations:** 1 Department of Engineering for Innovation, University of Salento, via Arnesano, 73100 Lecce, Italy; 2 Institute of Intelligent Systems for Automation, National Research Council, via G. Amendola 122/D, 70126 Bari, Italy; E-Mail: milella@ba.issia.cnr.it

**Keywords:** autonomous agriculture robotics, stereovision, self-learning classifier

## Abstract

Autonomous driving is a challenging problem, particularly when the domain is unstructured, as in an outdoor agricultural setting. Thus, advanced perception systems are primarily required to sense and understand the surrounding environment recognizing artificial and natural structures, topology, vegetation and paths. In this paper, a self-learning framework is proposed to automatically train a ground classifier for scene interpretation and autonomous navigation based on multi-baseline stereovision. The use of rich 3D data is emphasized where the sensor output includes range and color information of the surrounding environment. Two distinct classifiers are presented, one based on geometric data that can detect the broad class of ground and one based on color data that can further segment ground into subclasses. The geometry-based classifier features two main stages: an adaptive training stage and a classification stage. During the training stage, the system automatically learns to associate geometric appearance of 3D stereo-generated data with class labels. Then, it makes predictions based on past observations. It serves as well to provide training labels to the color-based classifier. Once trained, the color-based classifier is able to recognize similar terrain classes in stereo imagery. The system is continuously updated online using the latest stereo readings, thus making it feasible for long range and long duration navigation, over changing environments. Experimental results, obtained with a tractor test platform operating in a rural environment, are presented to validate this approach, showing an average classification precision and recall of 91.0% and 77.3%, respectively.

## 1. Introduction

Tractors are used for a variety of agricultural operations, including tilling, planting, weeding, fertilizing, spraying, hauling, mowing, and harvesting. Such versatility makes tractors prime targets for automation in order to improve productivity and efficiency, while preserving at the same time safe operations. Autonomous navigation in agricultural environments presents many challenges [1], due to the lack of highly structured elements in the scene that complicates the design of even basic functionalities. In addition to the geometric description of the scene, terrain typing is also an important component of the perception system. The ability to automatically recognize obstacles and different terrain classes would result in an enabling technology for autonomous navigation systems. Vehicles that can drive autonomously in outdoor environments have received increasing interest in recent years. Some notable examples can be found in the literature. On Mars, two robotic rovers have been exploring and collecting data since 2004. The Mars rovers, however, are carefully monitored and controlled; they cannot be considered as fully autonomous [2]. Another prominent example is the 2005 DARPA Grand Challenge [3], which featured fully autonomous vehicles racing over a 212 km desert course. Nevertheless, the Grand Challenge required vehicles to drive autonomously from waypoint to waypoint along a desert road: an arguably easier task than off-road navigation through arbitrary terrain. In the specific agricultural domain, various row guidance controls have been proposed using vision [4]; however they all rely on fixed landmarks and perform well in specific contexts. Although autonomous navigation has inspired decades of research, it still remains an open and active field of investigation. One of the critical challenges is accurate scene understanding to perform many important tasks, including environment segmentation and classification, mapping, and path planning.

This paper presents new sensor processing algorithms that are suitable for outdoor autonomous navigation. A three-sensor multi-baseline stereo camera is adopted that provides “rich” 3D data, *i.e.*, the raw output from the sensor is a 3D point cloud with associated color information. These algorithms have been developed and implemented within the project Ambient Awareness for Autonomous Agricultural Vehicles (QUAD-AV) funded by the ERA-NET ICT-AGRI action and aimed to enable safe autonomous navigation in high-vegetated, off-road terrain.

Scene understanding has been one of the goals of computer vision for decades. Recently, the application of statistical learning has given rise to new interest in this field [5]. Statistically trained models have an advantage over deterministic, hand-tuned systems, especially for complex scene analysis. Here, an adaptive self-learning framework using stereovision is proposed. Given 3D points, the system first maps them to cells and extracts geometric features of the points in each cell. Then, these features are used within a geometry-based classifier to label single cells in two broad categories, namely ground and non-ground patches. The ground class corresponds to points from the terrain, whereas the non-ground class corresponds to all other data, including points from above ground objects (*i.e.*, obstacles) or occluded areas, and poor stereo reconstructions. The classifier automatically learns to associate the geometric appearance of data with class labels during a training stage. Then, it makes predictions based on past observations classifying new observations. The geometry-based classifier also supervises a second classifier that uses color data to distinguish terrain subclasses within the broad ground class. Since the characteristics of the ground may change geographically and over time, the whole system is continuously retrained in every scan: new automatically labeled data are added to the ground model replacing the oldest labels in order to incorporate changes in the ground appearance.

The stereovision-based classifier leads to the following main advantages: (a) self-training of the classifier, where the stereo camera allows the vehicle to automatically acquire a set of ground samples, eliminating the need for time-consuming manual labeling, (b) continuous updating of the system during the vehicle's operation, thus making it adaptive and feasible for long range and long duration navigation applications, (c) extension of the short-range stereo classification results to long-range via segmentation of the entire visual image.

**Figure 1.**
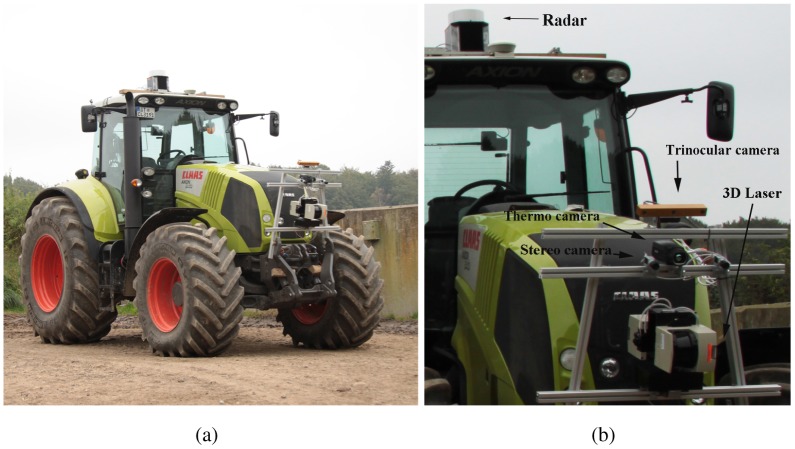
The tractor test platform employed in this research (**a**), and its sensor suite (**b**).

In this investigation, a PointGrey Bumblebee XB3 stereo system is employed. It consists of a trinocular stereo head, featuring two stereo configurations: a narrow stereo pair with a baseline of 0.12 m using the left and middle cameras, and a wide stereo pair with a baseline of 0.24 m using the left and right cameras. Additional technical details of the stereo system are collected in Table 1. The use of a trinocular configuration in place of a binocular one allows combining the advantages of two different baselines by the addition of one camera [6]. A narrow baseline increases the shared field of view of the two cameras, while yielding to shorter maximum range. Conversely, a larger baseline decreases the common field of view, but leads to higher maximum range and accuracy at each visible distance. By employing the narrow baseline to reconstruct nearby points and the wide baseline for more distant points, the trinocular system takes the advantage of the small minimum range of the narrow baseline, while preserving, at the same time, the higher accuracy and maximum range of the wide baseline configuration. The trinocular system is integrated with a CLAAS AXION 840 4WD tractor (see Figure 1), which has been employed for the testing and the field validation of the system. In Figure 1(b), the camera is visible, mounted on a frame attached to the vehicle's body and tilted forward of about 12° to minimize the field of view seeing the sky. The tractor's sensor suite is completed by a 3D Sick laser rangefinder, a 94-GHz frequency modulated continuous wave (FMCW) radar, and a thermal infrared camera [7].

The remainder of the paper is organized as follows. Section 2 reports related research in the field. The proposed self-learning framework is described in Section 3, whereas details of the statistical approach for ground classification are presented in Section 4. Sections 5 and 6 explain the geometry-based and color-based classifier, respectively. In Section 7, the system is validated in field experiments performed with the tractor test platform. Section 8 concludes this paper.

## 2. Related Work

Considerable progress has been made in recent years in designing autonomous, navigation systems for outdoor environments [8,9]. Progress has also been made in high-level scene analysis systems [10], with various application domains including on-road scene awareness [11,12], off-road rough terrain analysis for planetary rovers [13,14], off-road terrain classification for challenging vegetated areas [15,16], and agriculture [17–19]. In this section, research is organized by its learning strategy: deterministic (no learning), supervised, and self-supervised. Estimating the traversability of the surrounding terrain constitutes an important part of the navigation problem, and deterministic solutions have been proposed by many. However, deterministic techniques assume that the characteristics of obstacles and traversable regions are fixed, and therefore they cannot easily adapt to changing environments [14,20,21]. Without learning, such systems are constrained to a limited range of predefined environments. A number of systems that incorporate supervised learning methods have also been proposed, many of them in the automotive field and for structured environments (road-following). These include ALVINN (Autonomous Land Vehicle in a Neural Network) by Pomerlau [22], MANIAC (Multiple ALVINN Network In Autonomous Control) by Jochem *et al.* [23], and the system proposed by LeCun *et al.* [24]. ALVINN trained a neural network to follow roads and was successfully deployed at highway speed in light traffic. MANIAC was also a neural net-based road-following navigation system. LeCun used end-to-end learning to map visual input to steering angles, producing a system that could avoid obstacles in off-road settings, but did not have the capability to navigate to a goal or map its surroundings. Many other systems have been proposed in recent years that include supervised classification [16,25]. In Blas and Blanke [26], a combination of texture classification, mapping, and supervision is suggested for automatic baling. These systems were trained offline using hand-labeled data, thus limiting the scope of their expertise to environments seen during training. Only recently, self-supervised systems have been developed that reduce or eliminate the need for hand-labeled training data, thus gaining flexibility in unknown environments. With self-supervision, a reliable module that determines traversability can provide labels for inputs to another classifier. Using this paradigm, a classifier can be trained online using data from the reliable sensor. An example can be found in Milella *et al.* [27], where a visual classifier was trained by radar-driven labels. A self-learning ground classifier was discussed in [28], for radar image interpretation. Brooks *et al.* [29] proposed a self-supervised framework that predicts the mechanical properties of distant terrain based on a previously-learned association with visual appearance. Self-supervised learning also helped win the 2005 DARPA Grand Challenge: the winning approach used a probabilistic model to identify road surface based on color information extracted immediately ahead of the vehicle as it drives [3]. Stavens and Thrun [30] used self-supervision to train a terrain roughness predictor. An online self-supervised learning was used to train a lidar-based navigation system to predict the location of a load-bearing surface in the presence of vegetation [31].

In this paper, a self-learning framework using stereovision is proposed for ground classification. In this framework, a first classifier is used to classify the scene into ground and non-ground regions based on geometric data, and labels from this algorithm are used to automatically train a second classifier that performs terrain typing based on color information.

It should be also noted that most of the algorithms proposed in the literature assume that the world is flat [15,32], and obstacle detection amounts to identifying objects that “stick out” of the ground. However, in outdoor unstructured environments, this assumption is typically violated. In this work, ground plane reasoning is not explicitly needed and the system automatically adapts to the changing geometry of the terrain. In contrast to previous works that attempt to explicitly identify obstacles [16,33], the proposed approach aims to detect scene regions that are traversable-safe for the vehicle. This is a subtle, but significant difference; only those regions where there is evidence that it is safe are labeled as traversable, thereby avoiding both positive and negative obstacles without explicitly detecting them. An additional advantage of the proposed obstacle detection scheme is that the output traversability map can be directly employed by most grid-based planners [34].

## 3. Self-Learning Framework

**Figure 2.**
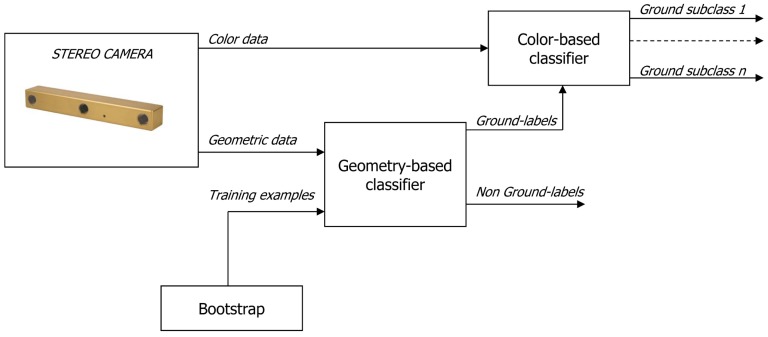
Architecture of the proposed self-learning framework. The training stage of the color-based classifier is supervised by the geometry-based classifier. The geometry-based classifier is initialized via a bootstrapping procedure and progressively adjusted during operation.

In the following, “self-learning classification” refers to automatic training of a stereovision-based ground classifier. Whereas in a traditional (*i.e.*, manually) supervised classifier a human user provides labeled training instances for each class of interest, in a self-learning framework another classification algorithm provides these training examples. In the context of this paper, a first broad classifier is proposed to identify ground patches based on geometric data. Then, color features associated with these ground patches are used to automatically train a second color-based classifier that performs terrain typing. Once the color-based classifier has been trained, it can identify instances of these terrain classes in the whole scene. Thus, the geometry-based and color-based classifier work in cascade according to the scheme shown in Figure 2. The training instances for the geometry-based classifier are also automatically produced using a rolling training set. The training set is initialized at the beginning of the robot's operation via a bootstrapping approach and progressively updated. Initially, the robot has no knowledge of the relationship between ground appearance and the ground class. The only underlying assumption to initialize the training set is that the vehicle starts its operation from an area free of obstacles, so that the trinocular system initially “looks” at ground only. Then, geometric features can be extracted from the 3D point cloud and associated with the ground class. When sufficient data is accumulated, the geometry-based ground classifier can be trained, and the ground class is related with point cloud properties. This allows the system to predict the presence of ground in successive scenes based on past observations. Such a classification task is generally difficult as the ground reconstruction is affected by a number of factors that are not easily measured and change over time, including the type of terrain surface, topology, lighting conditions, *etc.* This suggests that an adaptive approach is necessary in which the image interpretation changes as the vehicle moves and conditions vary. To this aim, the model (*i.e.*, the training window) is continuously updated using the most recent acquisitions.

In summary, self-learning systems eliminate the need for hand-labeled training data, thus gaining flexibility in unknown environments. The burden of hand-labeling data is relieved and the system can robustly adapt to changing environments on-the-fly.

## 4. Statistical Ground Classification

The ground modeling problem is formulated as a one-class classifier [35] for both the geometry-based and the color-based classifier. One-class classification methods are generally useful in two-class classification problems, where one class, referred to as the target class, is relatively well-sampled, while the other class, referred to as the outlier class, is relatively under-sampled or difficult to model. This is the case for our application where most of the patches belong to the ground with sparse instances of non-ground. Typically, the objective of a one class-classifier is that of constructing a decision boundary that separates the instances of the target class from all other possible objects. In our case, ground samples constitute the target class, while non-ground samples are regarded as the outlier class. In agricultural environments, non-ground samples are typically sparse; in addition, the variation of all possible non-ground classes is unlimited, which makes it difficult to model the non-ground class. In contrast, although it changes geographically and over time, the ground class is generally less variable than random objects. Furthermore, our objective is that of building a model of the ground. Therefore, it is reasonable to formulate the problem as a distribution modeling one, where the distribution to estimate is the ground class.

To model the ground, a multivariate Gaussian mixture is adopted. Specifically, Expectation Maximization (EM) with Bayesian Information Criterion (BIC) is used to fit the mixture to the available labeled ground feature vectors, defining adaptively the number of components. Then, a Mahalanobis distance-based classification approach is used to recognize whether a new pattern is an instance of the ground class following an outlier detection strategy.

### 4.1. Ground Modeling

The use of Gaussian Mixture Models (GMMs) is a well-established approach to clustering, since each cluster can be easily represented in a compact form using three main parameters: mean vector, covariance matrix and mass (*i.e.*, number of samples) of the cluster. EM is a common method to estimate the parameters of a GMM, however it requires a priori knowledge of the number of clusters in the data (*i.e.*, the number of components *k* of the Gaussian mixture). The choice of the optimal number of Gaussian components *k* is a critical problem in data clustering especially for online estimation problems, such as in terrain modeling applications [34]. On one hand, a small number of components may be unable to correctly identify non-homogeneous ground regions; on the other hand, a high value of *k* could lead to an over-fitting of the model of the training set with a loss of generalization power of the classifier. Furthermore, in autonomous exploration, a priori knowledge of *k* would entail the number of habitats to be known prior to training, which is not generally the case. In this work, EM and BIC is used to fit the data using a Gaussian Mixture Model, and estimate, at the same time, the optimal number of Gaussian components [36]. The implemented algorithm features a recursive procedure that starts with a single cluster assumption and iteratively applies EM with a growing number of clusters *k*. For each estimated GMM, the BIC coefficient is computed and the optimal number of clusters can be obtained as the value that minimizes the BIC coefficient.

Let *X_t_* be a *n* × *m* data table representing a sample of *x_i_* vectors with *i* = 1, 2,… *n*, each characterized by *m* traits: *X_t_* = {*x*_1_,…, *x_n_*}. These vectors constitute the training set at a given time *t* to construct the ground model as a mixture of multivariate Gaussians $G_{t}^{k}$ with *k* components, each one represented by *g_i_* = (*x̄_i_*, *S_i_*, *n_i_*), *i* = 1,2,…, *k*, where *x̄_i_* is the mean value, *S_i_* the covariance matrix, and *n_i_* the mass, *i.e.*, the number of feature vectors belonging to component *i*

(1) $$G_{t}^{k}=\{g_{1},g_{2},\ldots ,g_{k}\}.$$

In order to estimate $G_{t}^{k}$, a single Gaussian distribution is initially fit to the data using EM and the corresponding BIC is estimated as

(2) BIC=−2⋅lnL+flnn

where *f* is the number of free parameters (which in turns depends on the number of clusters *k* and on the number of feature variables *m*), and *L* is the maximum likelihood achievable by the model. The BIC aims to balance the increase in likelihood due to the use of a higher number of parameters, by introducing a penalty term that grows as long as the number of parameters is augmented. Using this criterion, the Gaussian mixture that minimizes the BIC for the given data set is looked for. Specifically, a single component (*k* = 1) is initially assumed; then, the number of Gaussian components is incremented one unit at a time and the associated BIC is calculated, until a maximum number *k_max_* is reached. An additional stopping criterion is added based on the mixing proportions of the components in the GMM: if the minimum mixing proportion of a component is less than a threshold (e.g., 10%), then iteration is stopped and only the GMMs estimated up to the previous iteration are retained. Finally, the GMM $G_{t}^{k*}$ with k* clusters is chosen, which minimizes the BIC, *i.e.*, the model corresponding to the highest Bayesian posterior probability. It should be noted that since traversable ground is reasonably characterized by almost homogeneous geometrical properties, the ground modeling problem for the geometry-based classifier can be simplified by assuming that the number of Gaussian components is at most one (*k_max_* = 1), as also demonstrated later in Section 5. Conversely, different terrain types within the ground class would most likely lead to a multi-modal distribution of color features; therefore, a mixture of Gaussian fitting problem with *k* > 1 has to be solved for the color-based classifier (see Section 6). A maximum value *k_max_* = 5 is fixed in this case based on physical considerations.

### 4.2. Model Update

The accuracy of a ground classifier greatly depends on the accuracy of the model adopted for the ground. The best choice of ground model is tightly connected with the environmental conditions in which the system is used. For example, a “static model”, built upon the initial geometric or color properties of the ground, could soon fail or give poor results because of changes in ground properties during vehicle travel. Here, an adaptive approach in the ground model building is proposed that allows the ground model to adjust online following a multi-frame approach without any a priori information. At the beginning of the robot's operation, the training set is initialized under the assumption that the vehicle starts from an area free of obstacles, so that the stereo camera “looks” at ground only. Then, the ground model is continuously updated as the vehicle moves: new ground feature vectors labeled in the most recent acquisitions are incorporated, replacing an equal number of the oldest ground instances. The size of the rolling window is kept constant (*i.e.*, *n* = 2500 in our case). Let *Z_t_*_+1_ = {*z*_1_, *z*_2_,…, *z_l_*} denote the set of *l* ground-labeled cells classified at time *t* + 1, then the training set for the next acquisition scan is obtained as

(3) $$X_{t+1}=\{(x_{l+1},\ldots ,x_{n}),Z_{t+1}\}.$$

### 4.3. Ground Classification

Given a new single observation *z*, where *z* is either a geometric feature vector in the geometry-based classifier or a color feature vector in the color-based classifier, its membership likelihood to the ground class can be obtained by calculating the Mahalanobis distance (MhD) with respect to all components *k* of the current ground model $G_{t}^{k}$

(4) $$d_{j}^{2}=(z-{\bar{x}}_{j})S_{j}^{-1}{\left(z-{\bar{x}}_{j}\right)}^{t}$$

for *j* = 1,…, *k* being *k* the number of available terrain models (see Equation (1)). The pattern is an outlier, *i.e.*, it is classified as a non-ground sample, if its squared Mahalanobis distance from the closest model is greater than a critical value $d_{\text{crit}}^{2}$. The delimiter (cutoff), *L_β_*, for outlying observations can be obtained as the quantile *β* of the *m* degrees of freedom chi-square distribution $\chi_{m}^{2}$ [28].

(5) $$L_{\beta }=\sqrt{\chi_{m;\beta }^{2}}$$

Any patch with minimum Mahalanobis distance *d* satisfying the inequality *d* ≥ *L_β_* may be suspected to be an outlier at significance level (1-*β*). Otherwise it will be labeled as a ground sample.

## 5. Geometry-Based Classifier

Geometry-based ground classification is a method for labeling observations based on their geometric properties. Specifically, the appearance of ground is constructed upon a set of geometric features that can be extracted from stereovision 3D reconstruction. The raw output of stereo processing is a cloud of range data points. Scene points reconstructed by both the narrow baseline and the wide baseline stereo configuration are fused in a unique point cloud and pre-processed using a statistical filtering approach. The resulting point cloud is successively divided into a grid of 0.4 m × 0.4 m terrain patches projected onto a horizontal plane. Geometric features are statistics obtained from the point coordinates associated with each terrain patch. The first element of the geometric feature vector is the average slope of the terrain patch, *i.e.*, the angle *θ* between the least-squares-fit plane and the horizontal plane. The second component is the goodness of fit, *E*, measured as the mean-squared deviation of the points from the least-squares plane along its normal. This is the same as the minimum singular value of the points' covariance matrix. The third element is the variance in the *z*-coordinate of the range data points, $\sigma_{z}^{2}$ The fourth component is the mean of the *z*-coordinate of the range data points, *z̄*. Thus, the geometric properties of each patch is represented as a 4-element vector $x=[\theta ,E,\sigma_{z}^{2},\bar{z}].$

**Figure 3.**
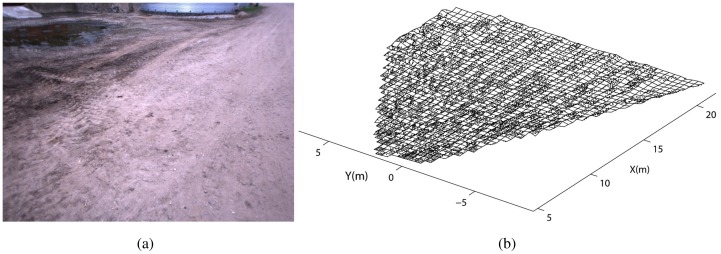
(**a**) A sample image acquired from a relatively flat area during the bootstrapping process to build the initial model of the ground class. (**b**) Associated 3D point cloud generated by stereovision processing and divided into a grid of 0.16 m^2^ cells.

**Figure 4.**
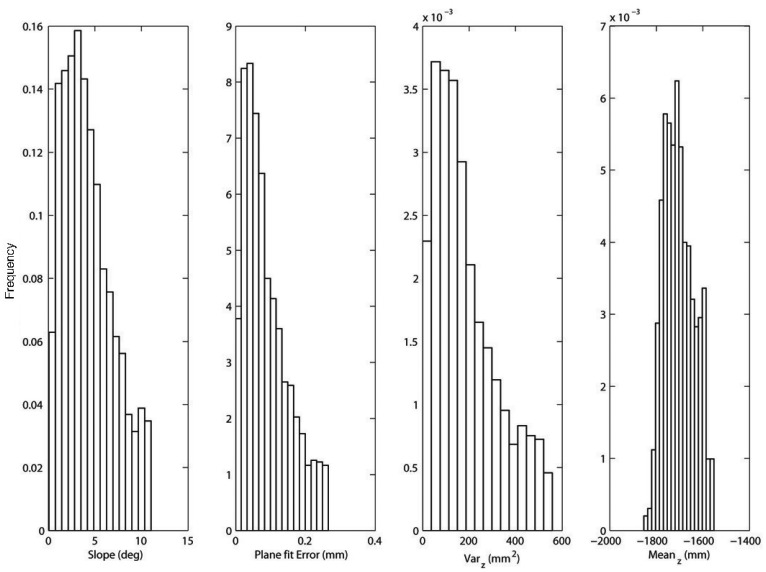
Normalized histograms of the distribution of the geometric features for a training window referring to relatively even agricultural terrain.

As an example, in Figure 3(a) a sample field scenario is shown. It refers to the bootstrapping process during which the geometric ground model is initialized at the beginning of the operation (refer to Section 3). The underlying assumption is that the robot faces relatively even terrain. The output of the stereovision processing is a 3D point cloud that is first divided into a grid of 0.16 *m*^2^ cells, as shown in Figure 3(b). Then, feature vectors can be extracted from each cell and the histograms of the distribution of the geometric features for the entire acquisition are shown in Figure 4. All four histograms exhibit an approximately unimodal distribution, which suggests that the ground model for the geometry-based classifier can be reasonably modeled using a single multivariate Gaussian (*i.e.*, a fixed value of *k* = 1).

## 6. Color-Based Classifier

Color data is directly available from the camera as red, green, and blue (RGB) intensities. However, illumination intensity affects all three values in a raw RGB representation, possibly leading to poor classification results. To reduce the effect of the overall illumination level, the so-called *c*_1_*c*_2_*c*_3_ color model is adopted [37]

(6) $$c_{1}=\arctan\left(\frac{R}{\max(G,B)}\right)$$

(7) $$c_{2}=\arctan\left(\frac{G}{\max(R,B)}\right)$$

(8) $$c_{3}=\arctan\left(\frac{B}{\max(R,G)}\right)$$

where R, G, and B are the pixel values in the RGB space. Thus, the color properties of each patch is represented as a 3-element vector *x* = [*c*_1_, *c*_2_, *c*_3_]. It should be noted that since there may be many pixels observed in each terrain patch, the overall estimate of the class likelihood, based on the pixels' color, is taken as the mean of the class likelihoods of the individual pixels. In the proposed self-supervised framework, color feature vectors associated with ground-labeled cells by the geometry-based classifier are automatically used for the color-based training.

**Figure 5.**
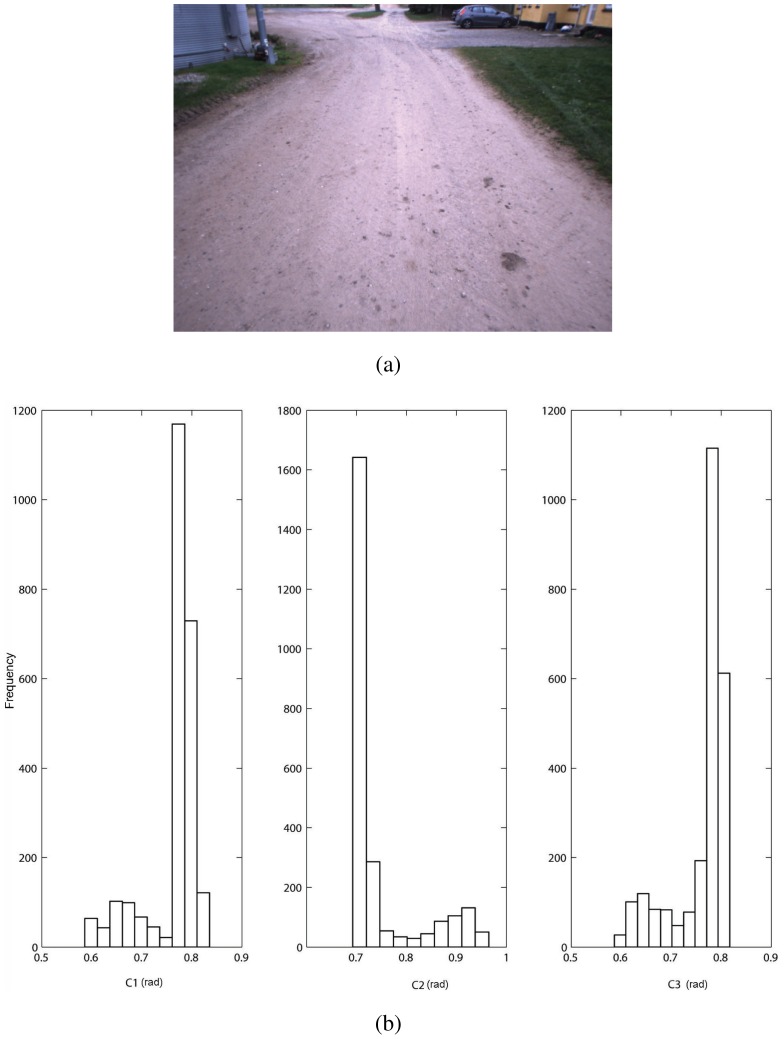
(**a**) A sample image acquired while the tractor is driving on a dirty road,(**b**) histograms of the distribution of the color features for the associated training window. The color-based classifier fits a mixture of Gaussians with *k* = 2 components, consistent with the presence of dirty road and grass in the scene.

As an example, Figure 5 shows a field scenario where the tractor drives along a dirty road delimited by side grassy areas that are relatively flat. Color feature vectors can be extracted from the training cells provided by the geometry-based classifier and the histograms of their distribution are shown in Figure 5(b). For the current training set, all three histograms suggest a multi-modal trend. When applying EM with BIC to fit the mixture of Gaussians to the available ground-labeled features, a number of components *k* = 2 is found, which is consistent with the presence of two main types of terrain in the scene, namely dirty road and grass.

## 7. Experimental Results

In this section, experimental results are presented to validate our approach for scene segmentation using stereovision data. The system was integrated with the experimental tractor (see Figure 1) and tested in a rural environment at a farm near Helsingør, Denmark. Various scenarios were analyzed including positive obstacles (trees, crops, metallic poles, buildings, agricultural equipment), negative obstacles (holes, ditches), moving obstacles (vehicles, people and animals), and difficult terrain (steep slopes, highly-irregular terrain, *etc.*). During the experiments, the tractor was driven by a human operator with a travel speed ranging between 2 and 15 km/h, as the onboard sensors acquired data from the surrounding environment. Then, the proposed classification framework was applied offline. Eleven experimental data sets were collected over the course of three days. Each data set consisted of a time series of stereo images and other sensors recorded during traverse of at least 250 m (up to 3 km). During the experiments lighting conditions ranged from diffuse lighting from an overcast sky to point lighting due to low, direct sunlight. For each data set, the tractor started its operations from an area that was clear of obstacles in order to initialize the ground model (refer to Figure 3) by acquiring a few scans (*s* = 3, in our case) during a short time interval (e.g., a 3 s window if a frame rate of 1 Hz was applied). After the training stage, the stereo classifier was able to predict the presence of ground in successive acquisitions.

### 7.1. Geometry-Based Classification

**Figure 6.**
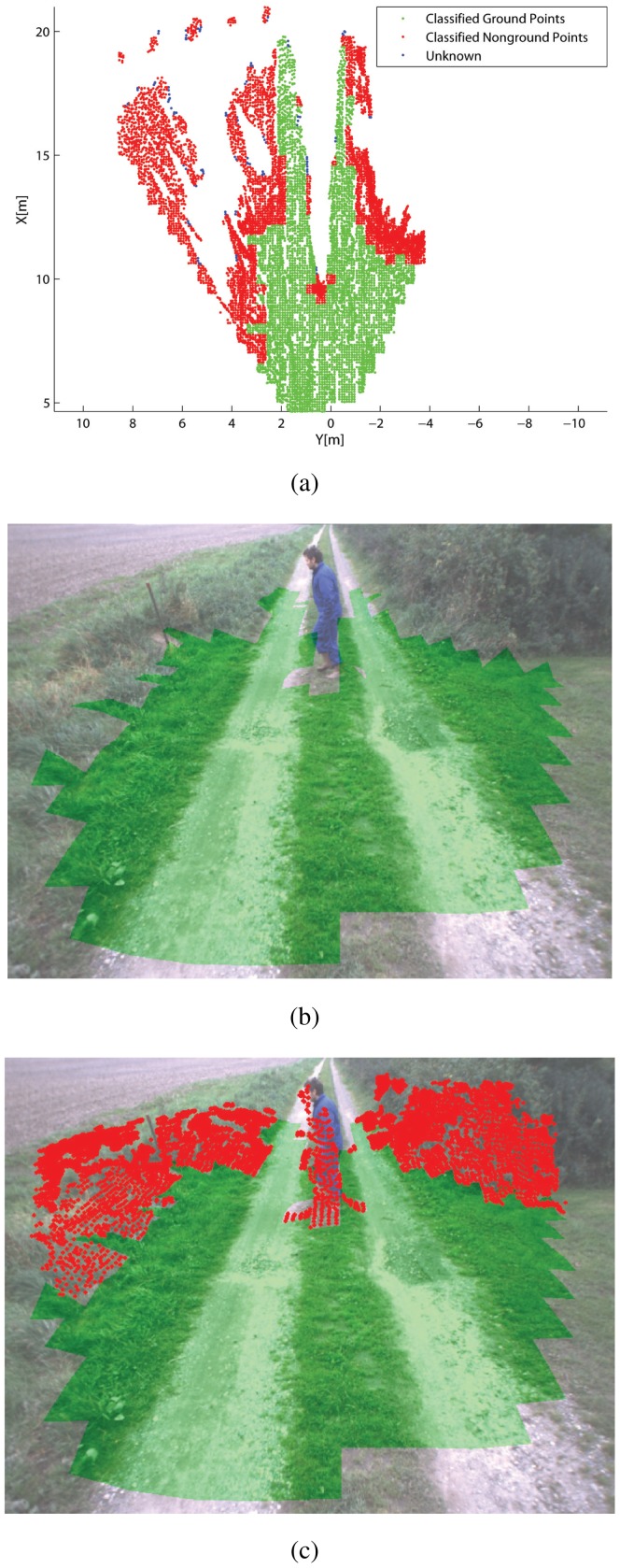
Results obtained from the geometry-based classifier for a scenario with a walking human operator: (**a**) classification of the raw 3D point cloud shown in the *x* − *y* plane of the tractor's reference frame. Green dot: classified ground. Red dot: classified non-ground. Blue dot: uncertain classification. Results projected over the original camera image: (**b**) only pixels associated with ground-labeled cells are marked using green, (**c**) 3D points associated with non-ground cells are also marked using red dots.

**Figure 7.**
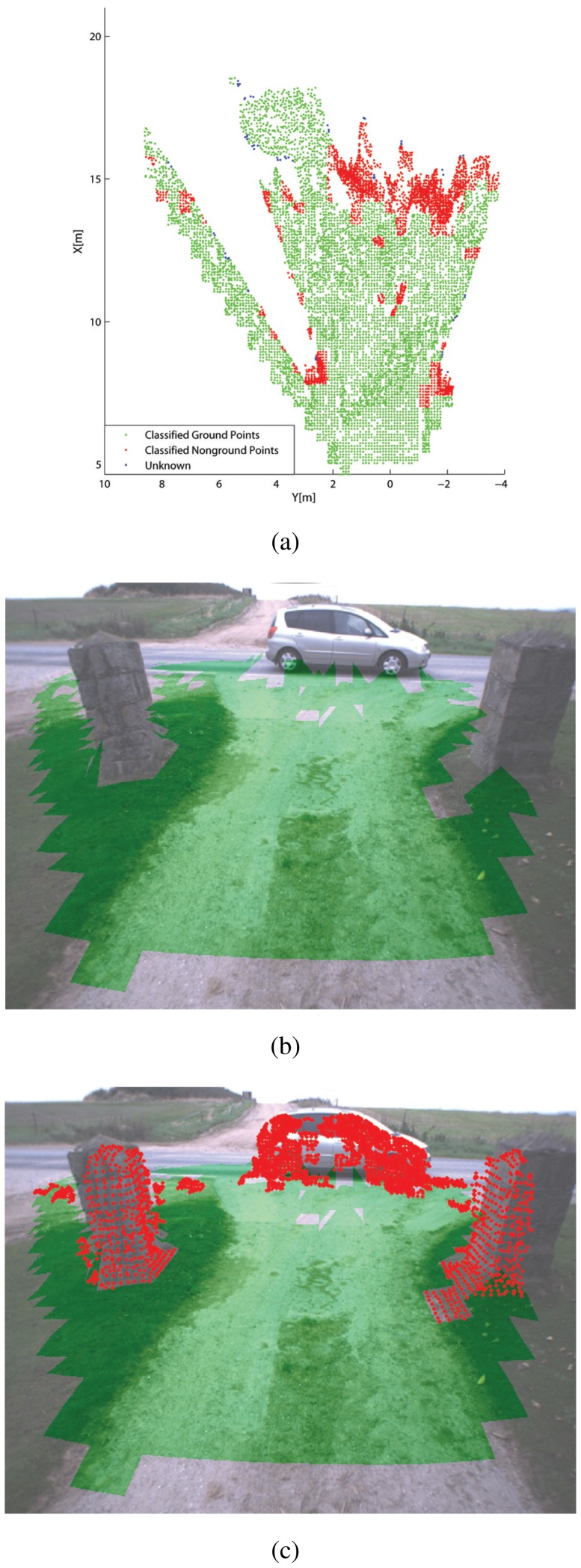
Results obtained from the geometry-based classifier for a scenario with static and dynamic obstacles: (**a**) classification of the raw 3D point cloud shown in the *x* − *y* plane of the tractor's reference frame. Green dot: classified ground. Red dot: classified non-ground. Blue dot: uncertain classification. Results projected over the original camera image: (**b**) only pixels associated with ground-labeled cells are marked using green, (**c**) 3D points associated with non-ground cells are also marked using red dots.

Figures 6–7 show some typical results obtained from the classifier during field experiments. A scenario where the tractor faces a human operator is shown in Figure 6. Figure 6(a) reports the results obtained from the classifier applied to the stereo-generated 3D point cloud. Points that belong to a cell labeled as ground are denoted by green dots, whereas points falling into cells marked as non-ground are denoted by red dots. Finally, blue dots refer to points that fall into sparsely populated cells (in our case with less than four points) that cannot be labeled by the classifier. In Figure 6(b,c), the results are projected over the image plane of the right camera for comparison and visualization purposes. Specifically, in Figure 6(b) only pixels associated with ground-labeled cells are marked in green, whereas Figure 6(c) shows as well the 3D points falling into cells labeled as non-ground that are overlaid over the original image using red dots. As can be seen from these figures, the classifier correctly detects the human obstacle and the irregular terrain along the dirty road. Figure 7 shows a different scenario where the tractor is about to cross a civil asphalt road that divides two crop fields. The vehicle stands in front of a narrow passage between two columns as a car drives rightward. The classifier successfully flags the ground and the different obstacles present in the scene.

### 7.2. Color-Based Classification

**Figure 8.**
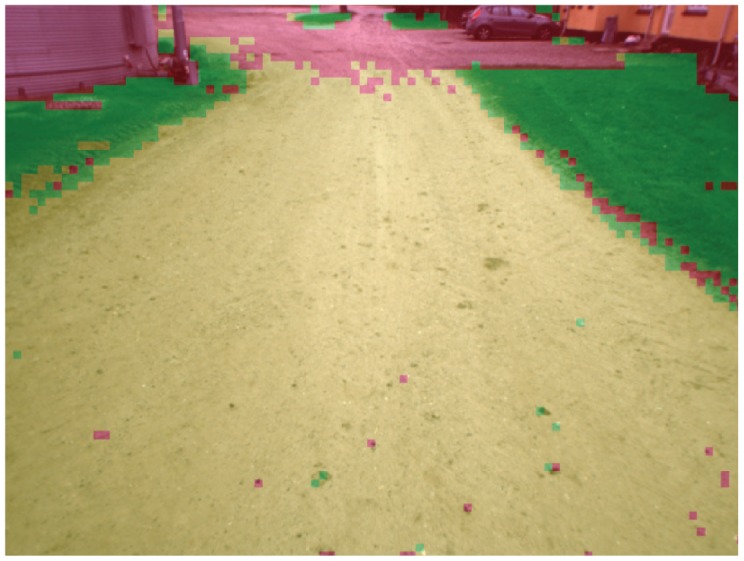
Results obtained from the color-based classifier as the tractor drives along a dirty road with grass on the side. Classification of the whole visual image. Yellow pixel: dirty road. Green pixel: grass. Red pixel: non-ground.

Figure 8 shows the results obtained from the color-based classifier for the scenario of Figure 5. As explained in Section 3, training examples are automatically provided by the geometry-based algorithm via a rolling training window that is progressively adjusted during operations. Once trained, the classifier can be applied to the entire visual scene. For this scenario two types of terrain have been found (*i.e.*, *k* = 2, as explained in Section 4.1). Pixels associated with the first type of terrain (dirty road) are marked in yellow, whereas pixels corresponding to the second type of ground (grass) are denoted using green. Finally, pixels labeled as non-ground are denoted using red. By continuously updating the training window, the system can adapt to new terrains within seconds.

**Figure 9.**
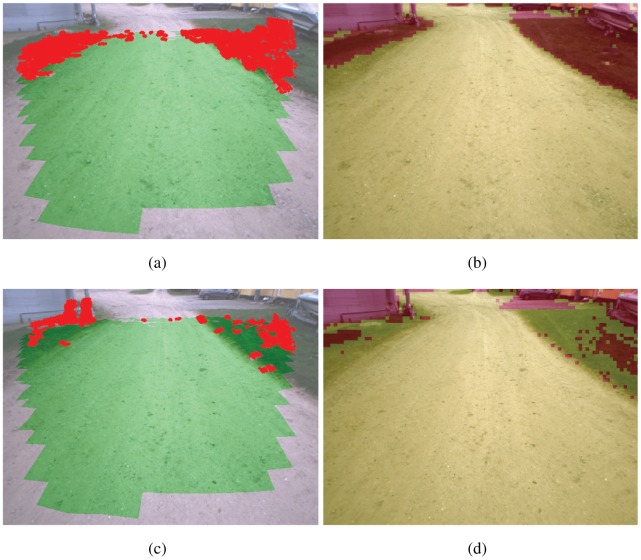
A sample sequence illustrating the rapid adaptation of the system to changes in the appearance of ground. (**a**) and (**c**) show the results of the geometric classification that supervises the training set of the color classification shown in (**b**) and (**d**). When the geometry-based classifier predominantly screens the dirty road, grass is not classified as drivable. As new instances of grass start populating the rolling training window, the classification changes.

The sequence in Figure 9 illustrates the adaptation at work: Figure 9(a,c) shows the training ground patches as obtained by the geometry-based classifier in two successive frames. Again, points that belong to a cell labeled as ground are denoted by green dots, whereas points falling into cells marked as non-ground are denoted by red dots. The 3D points are overlaid over the original visual image by perspective transformation. Figure 9(b,d) shows the results of applying the learned color-based classifier. As shown in Figure 9(a), no training instances of grass are initially provided by the geometry-based classifier. As a consequence, grass is not recognized by the system (Figure 9(b)). Nevertheless, the algorithm easily adapts as soon as new instances of grass are added to the training rolling window (Figure 9(c)) within less than a second while still correctly labeling obstacles present in the scene (Figure 9(d)).

In order to provide a quantitative evaluation of the system performance, the true positive and false positive rates, *i.e.*, the precision and recall, of the overall ground classifier were measured for a subset of images (*s_b_* = 40) taken from different data sets. This subset was hand-labeled to identify the ground-truth terrain class corresponding to each pixel. By assuming a typical significance level of 0.05 (*β* = 95% for the cutoff threshold expressed by Equation (5)), it resulted in an average precision of 91.0% and recall of 77.3%.

## 8. Conclusions

In this paper, a self-learning framework was described for scene segmentation by an autonomous agricultural tractor using trinocular stereovision. Experimental results obtained using a test platform in natural scenarios validated the proposed approach showing good classification performance. The classifier led to the following main advantages: (a) self-learning training of the classifier, where the trinocular system allows the vehicle to automatically acquire a set of ground samples, eliminating the need for time-consuming manual labeling, (b) continuous updating of the system during vehicle's operation, thus making it adaptive and feasible for long range and long duration navigation applications, (c) extension of the short-range stereo classification results to long-range via segmentation of the entire visual image. This technique can be successfully applied to enhance perception for autonomous off-road vehicles operating in agricultural settings.

## Figures and Tables

**Table 1.** Specifications of the stereovision system.

Camera	Model (baseline)	Image size (pixels)	Field of view	Optics	Range
Trinocular	Bumblebee XB3 (0.12/0.24 m)	1, 280 × 960	66° × 54°	focal length: 6 mm f2.5	2 to 22 m
